# Methodologies for determining staffing needs in healthcare: systematic literature review

**DOI:** 10.1093/eurpub/ckac131.260

**Published:** 2022-10-25

**Authors:** L Pirrotta, A Da Ros, P Cantarelli, N Bellè

**Affiliations:** Managemet and Healthcare Laboratory, Sant'Anna School of Advanced Studies, Pisa, Italy

## Abstract

The determination of staffing needs in healthcare is not just calculating the optimal number of professionals but is defining how the professional contingent accompanies the development of the healthcare organisation and of the population’s care needs. This research investigates the existence of a gold standard for determining health personnel requirements. We perform a systematic literature review to explore several approaches worldwide, examining a wide range of contextual variables, useful for the definition of an omni-comprehensive approach. A total of 557 articles was initially detected, then reduced to 57 after excluding everything not related to healthcare context and staff planning models. Results do not reveal a recognized standard for determining staffing needs. Approaches to the definition of staffing standards are mainly ex-ante (31%), based on the characteristics of specific models and organisational needs, or ex-post (62%), based on production analysis and historical trends. Most of these refer to the medical and nursing category (68.4%), while the minority proposes a multi-professional approach (17.5%). This review highlights innovative approaches based on algorithms which, starting from historical data, are adjusted by moderating key variables such as contextual factors, healthcare organisation models and professional attributes.

The review suggests:

1. Develop and share a unique tool for defining standards based on several variables that identify the characteristics of the context

2. Use up-to-date information flows and quality data

3. Consider a multi-professional approach

4. Adopt a long-term vision and continuous dialogue with the training process

It is clear the need to develop a tool for the definition of personnel requirements in line with internal and external changes in the health system. Therefore, such models need to account for an adequate number of variables, useful to identify the characteristics of the overall context.

**Key messages:**

• The development of staffing needs estimates must necessarily rely on a certain level of standardisation, but at the same time must take into account the variability characterising different contexts.

• In order to respond to recent demographic and epidemiological trends, it is crucial to include in the model skill mix and task shifting strategies involving health professionals as a whole.

